# Suggestions for the Busy Doctor

**Published:** 1892-07

**Authors:** 


### Suggestions for the Busy Doctor.

**Chloral in the Treatment of Boils.**—*Bull. Gen. de Therapeutique.* By M. Spehn.

The author recommends very highly as far superior to all other treatment the use of chloral externally in this troublesome class of affections. He directs that the boil be kept covered with a tampon of cotton-wool soaked in the following solution:

℞. Chloral hydrat........................℥iiss.

Aquæ

Glycerin........................aa f℥v.—M.

—*American Medical Magazine.*

---

**Hepatic Colic.**—Lemoine uses the following methods in the treatment of hepatic colic. If no vomiting is present, he has recourse to ethereal solutions or those containing chloroform, as follows:

R Syrupi acaciæ....................f℥iv.
Æther. sulphur....................f℥i.

Or

R Chloroform......................*m*xv.
Tincturæ myrhh..................*m*xv.
Mucilag. acaciæ....................f℥ii.
Syrup.......................f℥ifs.

A tablespoonful of either of these prescriptions every fifteen minutes.—*Review Therapeutique.*

---

**Treatment of Soft Chancres and Suppurating Buboes.**—Dr. Gamel, of Marseilles, France, praises camphorated carbolic acid in soft chancres. It hinders the development of phagedena and renders ordinary (soft) chancres simple wounds, to cicatrize in three days. Very extensive chancrous wounds healed rapidly under its influence. He employs the following formula:

R Crystalized carbolic acid.........gms. 10

(ʒijss).

Camphor........ ..........gms. 25

(ʒvi).

Mix and warm on a water bath.

This forms a limpid, syrupy liquid, similar to glycerine, and of a very agreeable odor. The dressings should be repeated twice a day. Clean the sore well with absorbent cotton and apply small pledgets dipped into this preparation. Keep this in place by a cotton dressing held in place by a salolized gauze strip. Suppurating buboes are treated by irrigation with a strong solution of carbolized water and dressed with phenolized camphor. Buboes which are slow to suppurate are easily managed with injections of iodoform in ether. Inject one cubic centimetre three times a week by means of a hypodermic syringe with a long needle.—*Lancet-Clinic.*

---

The Treatment of Diphtheria.—The following plan is pursued by Dr. Alexander Fulton (*Medical News*, April 23): Application to the diphtheritic patches, by means of a throat brush, of a strong solution of nitrate of silver (forty grains to the ounce). If practicable this is followed by a gargle, such as,

℞ Tinct. kino . . . . . . . . . . . . fl. ℥ij.

Glycerin . . . . . . . . . . . . . fl. ʒij.

Ol. eucalyptol . . . . . . . . . . .gtt. x.

M. Sig.—Teaspoonful in half ounce of water, as gargle.

Whether or not the gargle be used, the throat is dusted with the following:

℞ Hydrarg. chlor. corros . . . . . . . . gr. i.

Pulv. sulphuris . . . . . . . . . . . ʒi.

M. Sig.—Blow a "pinch" into the throat every four hours (with insufflator).

From the commencement of the disease Dr. Fulton gives internally (according to age):

℞ Pulv. potass. chlorat . . . . . . . ʒii.

Tr. ferri chloridi . . . . . . . . . . fl. ʒii.

Syr. limonis . . . . . . . . . . . . fl. ℥iii.

Ol. gualtheriæ . . . . . . . . . . . gtt. iii.

M. Sig.—Teaspoonful every two or three hours.

A mixture of tincture of iodine and camphorated oil is applied externally over the site of the tonsil. This method of treatment "has been successful in thirty-seven consecutive cases."—*The Atlanta Medical and Surgical Journal.*

---

Treatment of Erysipelas.—The treatment of erysipelas which has yielded the best results in our experience (*Ther. Gaz.*) is one which we recommended elsewhere, and which in each and every case proves to be singularly efficient.

It consists in washing the part which is affected with a solution of bi-chloride of mercury, in the strength of 1 to 10,000, and then thoroughly anointing the skin with an ointment of ichthyol of the strength of two drachms of ichthyol to the ounce of lanolin or benzoated lard. This having been applied, the part is protected by a layer of salicylated cotton and a gauze covering; or, if the disease involve the face, a piece of gauze can be used alone, with holes cut in it for the nose, eyes and mouth.

This line of local treatment in the early stages of the malady diminishes the pain and relieves the tension of the parts, at the same time seeming to limit the era involved. When the case is seen after the attack is well advanced, the same treatment rapidly reduces swelling and induration, and produces so rapid a change for the better that the patient soon recognizes the advances which he is making.

It is hardly necessary to add that full doses of tincture of chloride of iron should be given in such cases; as much as 20 or 30 drops every four hours if the disease is severe. Since this has been written the article of Klim has been published, which gives practically the same treatment.—*Medical Review.*

---

The Treatment of Gonorrhœa.—My treatment of gonorrhœa in all stages has for long been very monotonous. Almost without regard to stage or degree of severity, I prescribe the same remedies. I have long ago laid aside the traditions of my student days, which taught that salines only should be used in the acute stages, and that abortive plans were dangerous. I always use abortive measures, and mostly, I believe, succeed. At any rate, I never encounter ill consequences, and complications are rare. My prescription is a partnership of three different remedies, and it is, I believe, important that they should all be used. First an injection of solution of chloride of zinc, two grains to the ounce; next, sandalwood oil capsules; and lastly, a purgative night dose with bromide of potassium. The injection is used three or four times a day, the capsules (ten or twenty minims) taken three times a day. The ingredients of the night dose are three drachms of Epsom salts and a half drachm of bromide of potassium. It is, I believe, the action of the last named in preventing congestion of the parts which makes the abortive measures safe. Moderate purgation and entire abstinence from stimulants are essential. If the case is very acute, and attended by swelling of the corpus spongiosum, I sometimes prescribe tartar emetic or tincture of aconite, but it is very seldom, indeed, that these are necessary. If the patient be well purged, there is no risk whatever in an abortive treatment from the day that he comes under treatment. The risk of orchitis, prostatitis, cystitis, etc., comes in cases which have been allowed to develop rather than in those treated abortively. I should as soon think of delaying to use local measures in gonorrhœa as I should in purulent ophthalmia.—*Jonathan Hutchinson in Archives of Surgery.*

**The Value and Application of the Cystoscope.**—Meyer (*New York Medical Journal*) comes to the following conclusions as to the value and application of the cystoscope:

1. In all obscure reno-bladder diseases cystoscopy has to be practiced—if necessary, repeatedly—before operative interference for diagnostic purposes is resorted to.

2. There are a number of causes which make cystoscopy impracticable.

3. Cystoscopy is an easy and harmless examination, but its successful employment requires experience.

4. It should be performed as a *dernier ressort*, after all other well-known means for making a diagnosis have been exhausted.

5. If properly applied, cystoscopy will generally clear up an obscure disease of the bladder.

6. In most cases we can determine, with the help of electric illumination of the bladder, whether we have to deal with a disease of the bladder or of the kidneys.

7. We can thus find out whether there are two working kidneys, also whether only one of the two kidneys is diseased, or both.

8. We shall most probably soon be able, perhaps in the greatest majority of cases, after sufficient practical experience, and with the help of proper cystoscopic instruments designed for this purpose, to catheterize the ureters, and thus gather, in a bloodless manner, the urine from each kidney separately.

9. We can thus make out, in certain cases, by observing *the character* of the jets of urine, especially by *timing their frequency and duration* at the urethral orifices, whether the other kidney is doing the work of one which is diseased.

10. These facts will tend to make superfluous, in the majority of cases, at least, a preliminary suprapubic or perineal incision for diagnostic purposes, as well as a nephrotomy performed for determining the action of the other (not diseased) kidney. They greatly widen ann strengthen our means for determining the indication and prognosis of nephrectomy.

11. With the aid of Nitze's newest instrument, the operating cystoscope, we may look forward to being able to carry on intravesical treatment under the direct guidance of our eyes.—*Gaillard's Journal.*

[This surely is an age of "enlightenment" when one can light up the interior of the bladder and watch the distillation of urine from the kidneys, as it falls drop by drop into its receptacle.—ED.]

